# Recombinase-free cloning (RFC) protocol for gene swapping 

**DOI:** 10.22099/mbrc.2021.41923.1685

**Published:** 2022-03

**Authors:** Hai-Vy Vo-Nguyen, Thanh-Tan Nguyen, Quoc-Gia Mai, Thien-Thien Tran, Thuoc Linh Tran, Hieu Tran-Van

**Affiliations:** 1 Department Molecular and Environmental Biotechnology, Faculty of Biology and Biotechnology, University of Science, Ho Chi Minh City, Vietnam; 2 Laboratory of Biosensors, Faculty of Biology and Biotechnology, University of Science, Ho Chi Minh City, Vietnam; 3 Vietnam National University, Ho Chi Minh City, Vietnam

**Keywords:** Cloning, Recombinant DNA, Molecular biology, E.coli DH5α

## Abstract

Recombinant DNA technology has been playing the key role for a long time since its first beginning. DNA ligases have certainly contributed to the development of cloning techniques, as well as molecular study up to now. Despite being a prime cloning tool, DNA ligases still face some shortcomings which lead to their limit of use. Our study provided an improved method that simplified the basic restriction enzyme-based cloning (REC) by eliminating the ligation role, named recombinase-free cloning (RFC). This improved technique was designed with only one PCR reaction, one digestion reaction, and one temperature profile, which takes advantage of endogenous recombinase in *E. coli* host to create the target recombinant vector inside the cell. All purification steps were eliminated for effectively material- and time-saving. Five different clones were generated by RFC. This method showed relatively low efficiency yet successful at a range of 100% in every conducted trial with fragment sizes from 0.5-1.0 kbp. The RFC method could be completed within a day (about 9 hours), without the need of ligase or recombinase or purification steps, which significantly saved DNA components, materials as well as the time required. In conclusion, we expected to provide a more convenient cloning method, as well as enable faster generation of DNA clones, which would be well applied in the less equipped laboratories.

## INTRODUCTION

Molecular cloning, a term describing the generation of recombinant DNA molecules, has stimulated advances throughout gene research and related molecules as well. Since its beginning in the 1970s, recombinant DNA technology has reached significant progress in both expertise and practice, providing a fundamental yet powerful tool for DNA manipulation. A traditional cloning method, restriction enzyme-based cloning (REC), recognized as a standard laboratory technique for its simplicity and accessibility. REC relies on two crucial steps, enzymatic digestion and ligation to create vectors of interest [1]. Restriction enzymes are utilized as “scissors”, contributing components as digested vectors and genes with the same ligating ends, whereas ligases play the role of a “magic glue”, joining them into one combination. Together, these function-discrepant enzymes build a firm foundation for cloning technology. However, this REC excessively counts on enzymatic activity, which could give rise to decreased efficiency if one of these factors works ineffectively, especially for PCR products. While restriction enzymes and their buffers, as well as DNA ligase are quite stable as supplied [2, 3], buffers for DNA ligase containing ATP is not stable and decreased concentrations of ATP largely influence the ligation efficiency. Most of extensively used DNA ligases, specifically T4 DNA ligase, are ATP-dependent enzymes due to their ATP hydrolyzation during covalent linkage of the 5’-PO_4_ and 3’-OH groups. Hence, ligase buffers require subzero-storage condition, considerably raising the cost and limiting availability in developing countries. This is one of the most influential drawbacks of the prime tool for molecular biology. Ligases have been keeping the key role for so long, then what will happen if this powerful tool is eliminated in cloning procedure? Many efforts have been carried out to solve the question: MCT cloning [4], ligation-independent cloning [5], restriction free cloning [6], etc. In addition, homologous recombination is also a considerable replacement, in which commercial recombinase [7, 8] or megaprimers [9] are exploited. However, these approaches employ special enzyme treatment like T4 DNA polymerase [5] or *Dpn*I [4, 6, 9] and multiple PCR reactions [4], some need expensive enzymes which leads to their limit of use [7, 8]. A variation of this approach is seamless ligation cloning extract (SLiCE), an innovative seamless DNA cloning technique utilizing *in vitro* homologous recombination activities in *Escherichia coli* cell lysates to construct recombinant plasmids [10, 11], which helped solve the expensive recombinase matter. A homemade version of SLiCE was introduced and considered to eliminate the cost disadvantage of commercial version [12, 13]. However, this homemade SLiCE required a bunch of preparations with many procedures, which was not time-saving. Another remarkable ally that needs mentioning is recombinase-free cloning (RFC). This method employed the endogenous recombinase in *E. coli* host, even with RecA-deficient DH5 strain [14-16], to create the target recombinant vector inside the cell. It seemed to resolve the time and cost problems over others yet not the efficiency.

**Figure 1:**
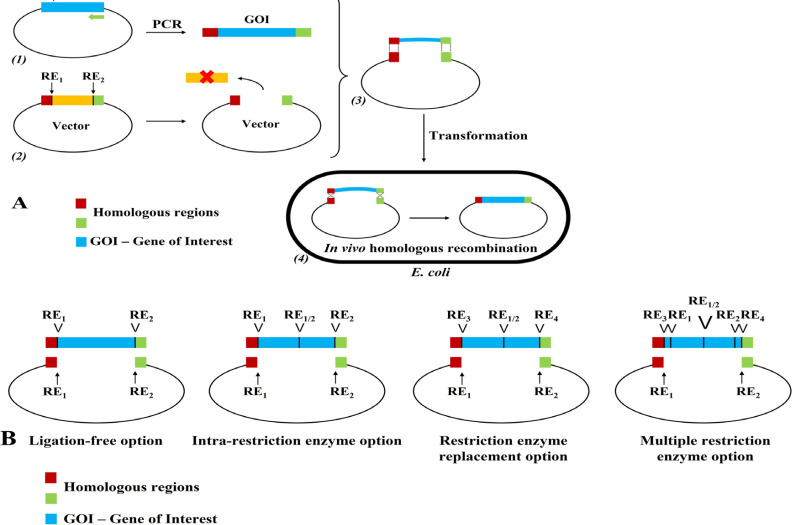
General scheme for RFC method (A) and possible options (B). (1), Amplification of target gene; (2), Enzymatic digest of vectors; (3), Mixture for a series of temperature changes; (4), *In vivo* homologous recombination in *E.coli *cells. Arrows indicate restriction enzyme digested vector, Checks (V) indicate restriction enzyme location

In this study, we introduced an improved cloning technique, which was expected to be simpler and time-saver. This method required only one PCR reaction [4, 17, 18], one restriction enzymatic digestion and no expensive recombinase or any complicated procedure [7, 8, 10, 12, 13], which takes advantage of endogenous recombinase in *E. coli* host to create the target recombinant vector inside the cell. The RFC method is employed when restriction enzymes cannot be used to create ligated ends due to the endogenous presence of respective restriction enzymes in the gene of interest, or in order to generate new restriction enzymes fringing the target gene, or in order to establish a ligase-independent strategy. The construction of recombinant vectors utilizing RFC included four steps, described in Figure 1. 

## MATERIALS AND METHODS

**Bacterial strains and plasmids:** *E. coli* DH5α [F- end A1 hsdR17 (rk-/mk-) supE44 thi λ-recA1 gyrA96 ΔlacU169 (φ80 lacZ ΔM15)] and *E. coli* BL21 (DE3) (F+ ompT hsdSB (rB-mB-) gal dcm (DE3) were used as host strains for cloning and protein expression, respectively. The pBAD plasmid was used as a cloning vector. All strains and plasmids were provided by the Department of Molecular and Environmental Biotechnology, University of Natural Sciences, VNU-HCM, Vietnam.

**Primer design and Gene of interest amplification:** In general, the gene of interest needed to be amplified in the first step (Fig. 1A1). This amplification required a pair of designed primers which not only targeted the sequence of interest but also had at least 15-30 bp overlap recognition at their ends. The overlap recognition contained homologous regions to the cloning sites on the vector of choice (red and green parts in Fig. 1A1). Therefore, the PCR products comprised targeted gene flanked by vector homologous region at the two ends. Gene of interest was amplified using MyTaq RedMix (Bioline) with designed primers. Amplification reactions were performed in a total volume of 50 µL. The PCR products were confirmed on a 1.5% agarose gel. 

**Enzymatic digestion of cloning vectors:** In the second step, the cloning vector was digested with restriction enzymes to create linear fragments (Fig. 1A2). 1000 ng of vectors for cloning were digested with at least two restriction enzymes to lower the self-ligation effect as well as improve the cloning efficacy. Possible options for vector digestion were showed in Figure 1B. For examples, when either one or both restriction enzymes (RE) located within the gene of interest (GOI) making REC impossible, intra-restriction enzyme option offered an alternative by adding homologous regions to flank the REs. This would leave the REs intact. Another was restriction enzyme replacement option in which the ezyme(s) that cut the GOI was substituted by any RE of choice. Finally, in multiple restriction enzyme option, many REs could be inserted within the homologous regions. Digestion reactions were performed in a total volume of 20 µL. The digested products were confirmed on a 1.5% agarose gel and inactivated at suitable conditions. 

**Temperature profile for RFC:** After the enzymatic digestion, 8 µL linear fragments were mixed with 6 – 8 µL of PCR product from step 1, and the mixture was incubated in PCR cycler with the following temperature profile (72^0^C in 2 minutes, 65^0^C in 2 minutes, 58^0^C in 30 minutes, 10^0^C in at least 10 minutes) [19] (Fig. 1A3). At 72^0^C, the mixture would be slightly denatured to completely remove all unspecific annealing or digested residues from the vectors. At 65^0^C was a step for annealing of the target gene-containing fragments and the linear vectors to create a loosely-knitted construct by hydrogen bonds. These weak interactions were maintained during 58^0^C step. The mixture then was cooled down to 10^0^C to keep the pre-recombinant vectors intact. The hybridized products were stored on ice until transformation. From the second step, all reactions were performed in one single 0.2 mL PCR tube. All purification steps were eliminated.

**Figure 2:**
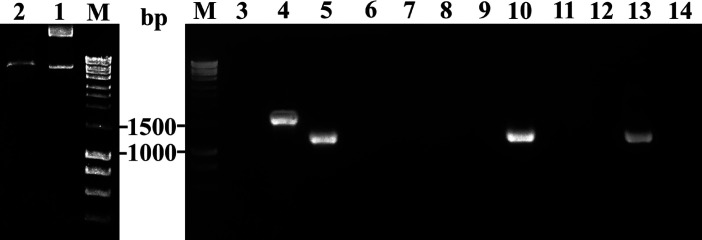
Enzymatic digest of vectors and verification of recombinant plasmid by PCR colonies of *E. coli* DH5α. M, DNA marker 1 kb; 1, original vector; 2, digested vector; 3, negative control; 4, parental vector; 5-14, screened colonies; 5, 10, 13, positive clones

**Bacterial transformation:** Finally, 10 µL of the hybridized products was transformed into competent *E. coli* DH5α cells, in which the homologous recombinant sites would be repaired *in vivo* (Figure 1A4). Cells were incubated on ice for 10 minutes, heat shocked for 90 seconds at 42^0^C, and then incubated on ice for 10 minutes. LB broth was added to the cells and incubated for 30 minutes at 37^0^C in shaking condition. The culture was then centrifuged and plated on selective-factor-containing LB agar. Positive clones were screened using colony PCR (Fig. 2). 

**Expression of recombinant protein:** Target vector collected from positive clones was transformed into competent *E. coli* BL21 (DE3) cells. Vector-carrying colonies were inoculated in shaking-LB-ampicillin tubes and allowed to grow at 37^o^C in overnight. Then, sub-culturing at 1:10 (v/v) and inoculating at 37^o^C until OD_600_ reached 0.8–1.0. Induction for protein expression was conducted immediately with suitable concentration of inducer and under proper conditions. Protein-expressing cells were collected and processed in PBS (pH 7.4) to obtain proteins in total, soluble, and insoluble phases. SDS-PAGE and Coomassie Brilliant Blue stained were utilized for analyzing the expression result. Finally, the targeted proteins were confirmed by Western Blot using specific antibodies for c-Myc tag (Thermo) and HRP-conjugated goat anti-mouse IgG-HRP (Proteintech).

## RESULTS AND DISCUSSION

Here was an example of cloning *chitosanase* gene into pBAD-Ag85 vector (Fig. 2). Designed primers were listed in Table 1. pBAD-Ag85 vector with the size of 5589 bp was digested with *Sal*I*/Pst*I (Thermo) to create linear fragments (Fig. 2A, lane 2), which qualified for annealing step of pBAD and *chitosanase* gene. Conventional PCR was performed to amplify the gene of interest (data not shown) as well as to verify the positive colonies (Fig. 2B). There were three positive colonies above 22 colonies screened, confirmed by PCR colonies with BAD-F/385R primers. *Chitosanase *gene has a theoretical size of 771 bp when being amplified by its specific primers, and the sequence from the gene to BAD-F site is about 488 bp, which meant the PCR product would have the size of about 1259 bp. As shown in Figure 2B, the screened bands lied between the 1000 bp and the 1500 bp band of the marker, equivalent to the predicted size of the targeted clone. The parental vector carried Ag85 gene with the size of 1181 bp, which appeared at about 1790 bp when PCR with BAD-F/BAD-R primers, equivalent to the size compared to the marker bands. Although this was quite a low efficacy, it did contribute to a huge effort of saving. No point mutations were detected after DNA sequencing for all cloned genes (data not shown) and expressed chitosanase showed on SDS-PAGE analysis.

**Table 1:** Example primers

**Primer**	**Sequence** **(5’-3’)**	**Amplicon Size (bp)**	
384F	GTCAAAAAACAGGTGTCGACgcgggactgaataaagatca		771
385R	AACAGCCAAGCTTCGAATTCtcacagatcctcttctgaga		

**Figure 3:**
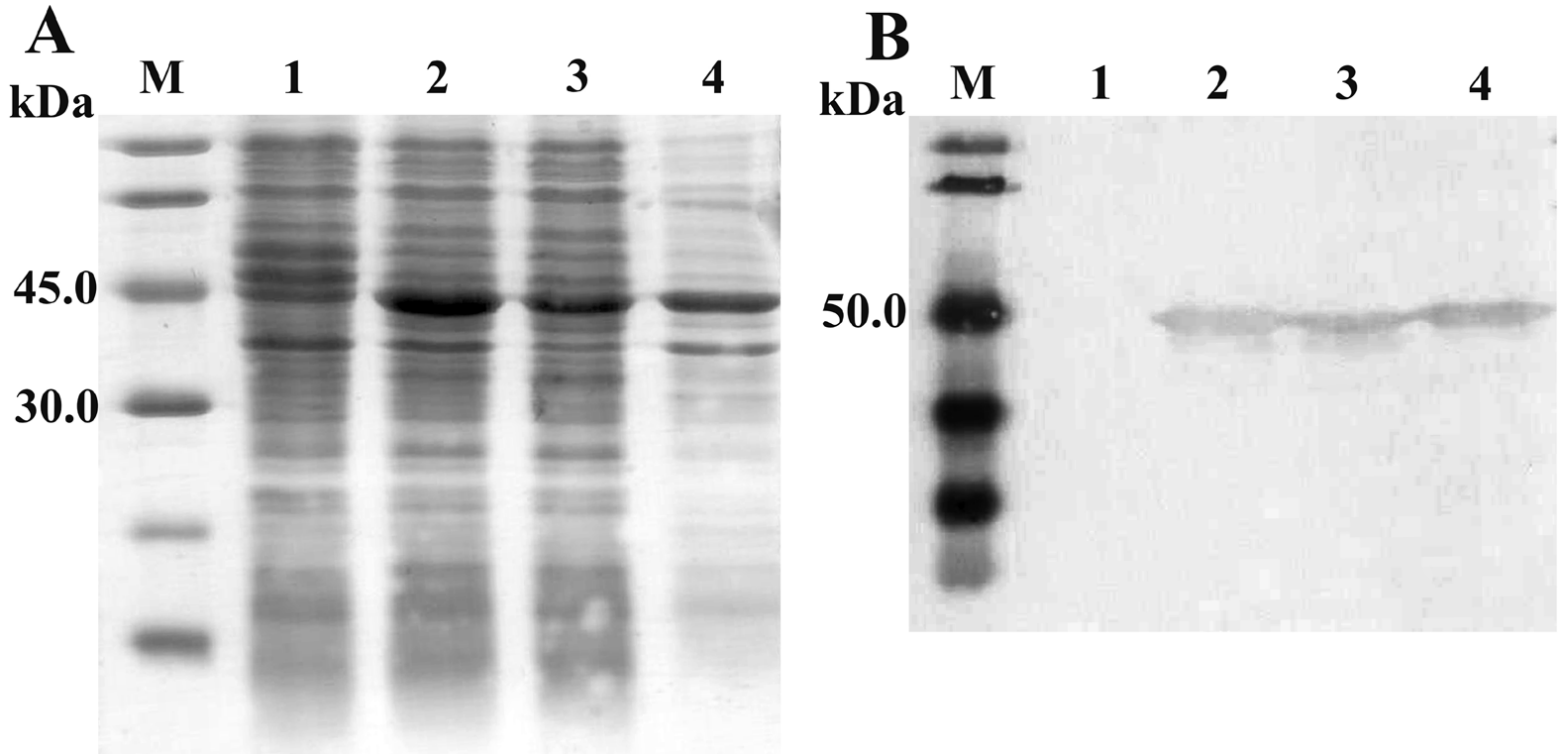
Expression of chitosanase analyzed by Coomassie Brilliant Blue staining (A) and confirmed by Western Blot (B). M, protein marker; 1, *E.coli* BL21(DE3)/pBAD-*chitosanase* (-L-arabinose); 2-4, *E.coli* BL21(DE3)/pBAD-*chitosanase* (+L-arabinose); 2, total phase; 3, soluble phase; 4, insoluble phase

The pBAD-*chitosanase* vector was transformed into *E. coli* BL21(DE3) cells for protein expression. Positive clones were induced by L-arabinose (0.7 mg/mL) to produce recombinant proteins. After induction, protein-expressing cells were lysed and analyzed by 15% gel SDS-PAGE and stained with Coomassie Blue. The observed bands on gel showed overexpression of one band at about 45 kDa (lane 2-4; Fig. 3A) compared to the marker bands, which were exact the predicted sizes of chitosanase (44 kDa). This band was also available in lane 2-4, Figure 3B, indicating that chitosanase expressed both in soluble and insoluble fractions. Meanwhile, there was no overexpression band detected in the negative control (lane 1, Fig. 3).

This method was performed in a relatively short time, consisting of one PCR reaction (about 2 hours), one enzymatic digest (about 4-6 hours, but this could be proceeded during PCR reaction), a temperature incubation (nearly 1 hour), followed by bacterial transformation (about 2 hours). Thus, the RFC method could be completed within a day (about 9 hours), without the need of ligase or recombinase or purification steps, which significantly lowered the costs. The efficiency of the RFC method was qualified by five genes with sizes ranging from 0.5 to nearly 1.0 kbp were cloned into different vectors (Table 2). With this RFC method, there was 100% of success, although the efficiency was quite low.

**Table 2:** Tested clones using the RFC cloning method

**Gene**	**Size (bp)**	**Cloning vector**
*pep1-f18s*	553	pY3T57
*gfp*	751	pET-hFc
*chitosanase*	771	pBAD
*pep1-f18a*	790	pYES2
*agglutinin*	960	pFRP1432

Another successfully performed example was GFP-hFc [20], in which RFC method was conducted in a brief way, which shortened the temperature incubation into only 30-minute step. In fact, after multiple tests, we could conclude that temperature incubation would be more efficient in longer genes (nealy 1.0 kbp). With shorter ones (0.5-0.7 kbp), a brief incubation should be utilized for time-saving.

The discovery of DNA ligases in 1967 was considered as a watershed occasion, which introduced a prime tools for molecular biology [21]. DNA ligases are indispensable for DNA replication and repair in all living things, also a crucial agent facilitating the development of molecular cloning and many subsequent segmentations of biotechnology. In our present study, we documented for the first time a cloning strategy which helped remove DNA purification step and lower the loss of processed DNA, reduce chemicals and time, and totally eliminate DNA ligases as well as other special enzymes in cloning. According to our achievements, further investigations should be proceeded to enhance and complete this technique. More fragment sizes and multiple fragments would be tested (under 0.5 kbp and over 1.0 kbp) to emphatically confirm the method’s efficiency. 

## Conflict of Interest

The authors declare that they have no competing interests

## References

[1] Vo-Nguyen H-V, Nguyen TT, Thi Vu HT, Thi Nguyen TT, Hoang QC, Tran TL (2021). Recombinant human SCARB2 expressed in Escherichia coli and its potential in enterovirus 71 blockage. Iran J Sci Technol Trans A.

[2] March JB, J Clark (2000). Enzymes by post—restriction enzyme stability. Nat Biotech.

[3] Youngblom J (2003). Extended stability of Taq DNA polymerase and T4 DNA ligase at various temperatures. BioTechniques.

[4] Li WY, Changjun L, Wu L, Wu JF, Yin XN, Deng KH, Zhang DY, Er M (2018). MCT cloning: a seamless cloning strategy for inserting DNA fragments. Biotechnol Biotechnol Equipment.

[5] Aslanidis C, De Jong PJ (1990). Ligation-independent cloning of PCR products (LIC-PCR). Nucleic Acids Res.

[6] Van Den Ent F, Löwe J (2006). RF cloning: a restriction-free method for inserting target genes into plasmids. J Biochmem Biophys Methods.

[7] Karimi M, D Inzé, A Depicker (2002). GATEWAY vectors for Agrobacterium-mediated plant transformation. Trends Plant Sci.

[8] Zhu B, Cai G, Hall EO, Freeman GJ (2007). In-Fusion™ assembly: seamless engineering of multidomain fusion proteins, modular vectors, and mutations. Biotechniques.

[9] Mathieu J, Alvarez E, Alvarez PJ (2014). Recombination-assisted megaprimer (RAM) cloning. Methods X.

[10] Zhang Y, Werling U, Edelmann W (2012). SLiCE: a novel bacterial cell extract-based DNA cloning method. Nucle Acids Res.

[11] Motohashi K (2015). A simple and efficient seamless DNA cloning method using SLiCE from Escherichia coli laboratory strains and its application to SLiP site-directed mutagenesis. BMC Biotech.

[12] Okegawa Y, Motohashi K (2015). A simple and ultra-low cost homemade seamless ligation cloning extract (SLiCE) as an alternative to a commercially available seamless DNA cloning kit. Biochem Biophys Rep.

[13] Motohashi K (2017). Evaluation of the efficiency and utility of recombinant enzyme-free seamless DNA cloning methods. Biochem Biophys Rep.

[14] Lovett ST, Hurley RL, Sutera VA, Aubuchon RH, Lebedeva MA (2002). Crossing over between regions of limited homology in Escherichia coli: RecA-dependent and RecA-independent pathways. Genetics.

[15] Dutra BE, VA Sutera, ST Lovett (2007). RecA-independent recombination is efficient but limited by exonucleases. Proc Natl Acad Sci USA.

[16] Jain K, Wood EA, Romero ZJ, Cox MM (2021). RecA‐independent recombination: Dependence on the Escherichia coli RarA protein. Mol Microbiol.

[17] Jacobus AP, Gross J (2015). Optimal cloning of PCR fragments by homologous recombination in Escherichia coli. PLoS One.

[18] Wang Y, Liu Y, Chen J, Tang MJ, Zhang SL, Wei LN, Li CH, Wei DB (2015). Restriction-ligation-free (RLF) cloning: a high-throughput cloning method by in vivo homologous recombination of PCR products. Genet Mol Res.

[19] Liang J, Liu Z, Low XZ, Ang EL, Zhao H (2017). Twin-primer non-enzymatic DNA assembly: an efficient and accurate multi-part DNA assembly method. Nucleic acids research.

[20] Nguyen TT, Vo-Nguyen HV, Tran-Van H (2021). Prokaryotic expression of chimeric GFP-hFc protein as a potential immune-based tool. Mol Biol Res Commun.

[21] Gellert M (1967). Formation of covalent circles of lambda DNA by E coli extracts. Proc Natl Acad Sci USA.

